# Disentangling the role of wild birds in avian metapneumovirus (aMPV) epidemiology: A systematic review and meta‐analysis

**DOI:** 10.1111/tbed.14680

**Published:** 2022-08-22

**Authors:** Giulia Graziosi, Caterina Lupini, Elena Catelli

**Affiliations:** ^1^ Department of Veterinary Medical Sciences University of Bologna Ozzano dell'Emilia, Bologna, BO Italy

**Keywords:** avian metapneumovirus, meta‐analysis, seroprevalence, systematic review, viroprevalence, wild birds

## Abstract

Given the avian metapneumovirus (aMPV) disease burden in poultry worldwide and the evidence of a possible role played by wild birds in the virus epidemiology, the present study summarizes aMPV serological and molecular data on free‐ranging avifauna available in the literature by conducting a systematic review and meta‐analysis. A computerized literature research was performed on PubMed, Scopus, CAB Direct and Web of Science to identify relevant publications across the period 1990–2021, along with the screening of reference lists. A random‐effect model was applied to calculate pooled prevalence estimates with 95% confidence intervals. The inconsistency index statistic (*I* ^2^) was applied to assess between‐study heterogeneity. Subgroup analyses for molecular studies only were performed according to geographical area of samplings, taxonomic order, genus and migration patterns of the birds surveyed. A total of 11 publications on molecular surveys and 6 on serological ones were retained for analysis. The pooled molecular prevalence was 6% (95% CI: 1–13%) and a high between‐study heterogeneity was detected (*I* ^2^ = 96%, *p* < .01). Moderator analyses showed statistically significant differences according to geographical area studied, taxonomic order and genus. Concerning serological prevalence, a pooled estimate of 14% (95% CI: 1–39%), along with a high between‐study heterogeneity, was obtained (*I* ^2^ = 98%, *p* < .01). Moderator analysis was not performed due to the scarcity of eligible serological studies included. Overall, molecular and serological evidence suggests that some wild bird taxa could play a role in aMPV epidemiology. Particularly, wild ducks, geese, gulls and pheasants, according to scientific contributions hereby considered, proved to be susceptible to aMPV, and due to host ecology, may act as a viral carrier or reservoir. Further surveys of wild birds are encouraged for a better comprehension of the poultry/wild bird interface in aMPV epidemiology and for better characterizing the virus host breadth.

## 1 INTRODUCTION

### 1.1 Rationale

Avian metapneumovirus (aMPV) is an enveloped negative‐sense RNA virus included in the genus *Metapneumovirus* of the Pneumoviridae family (Rima et al., 2017). aMPV is directly transmitted and causes upper respiratory tract disease and reproductive disorders in turkeys, chickens, domestic ducks and Guinea fowls, with subsequent economic and welfare issues for the worldwide poultry industry (Cecchinato et al., 2018; Rautenschlein, 2020).

To date, four aMPV subtypes (A, B, C and D) have been recognized according to genetic or antigenic characteristics (Bäyon‐Auboyer et al., 2000; Juhasz & Easton, 1994; Seal, 2000). Recent detections of phylogenetically distinct aMPV strains in North America suggest the existence of additional viral subtypes. Particularly, a divergent aMPV strain was detected in a great black‐backed gull (*Larus marinus* linnaeus, 1758) (Canuti et al., 2019) and another in a monk parakeet chick (*Myiopsitta monachus* boddaert, 1783) (Retallack et al., 2019).

With respect to viral spreading, aMPV‐A and B are distributed almost worldwide and are especially found in Asia, Africa, Europe and South America; aMPV‐C, which has been further recognized in a North American and a Eurasian genetic lineage, has occurred in the United States (North American lineage) (Senne et al., 1997) and in France and China (Eurasian lineage) (Sun et al., 2014; Toquin et al., 1999, 2006; Wei et al., 2013). aMPV subtypes also differ in terms of host breadth. aMPV‐A, B and C (North American lineage) mainly infect turkeys or chickens, while the Eurasian subtype C lineage infects ducks, and the subtype D (detected only once in France) turkeys (Rautenschlein, 2020). However, experimental evidence suggests that the subtypes’ host‐range may be more intricate (Brown et al., 2019).

Since aMPV's first appearance in South Africa in the late 1970s (Buys & du Preez, 1980), its origin and subsequent spread worldwide have been repeatedly ascribed, among other factors, to wild birds and to their migratory movements along their natural flight paths (Jones, 1996; Panigrahy et al., 2000; Shin et al., 2002). Particularly, aMPV's sudden appearance in Minnesota (USA), a state with a large migratory bird population, strongly contributed to this hypothesis (Cook, 2000b). Further evidence of a seasonal pattern observed during aMPV outbreaks in North American turkeys and viral detections of aMPV in wild birds outside endemic territories, suggested wild species as a viral carrier or reservoir host (Shin et al., 2002; Turpin et al., 2008). Additional viral or serological evidence of aMPV occurrence in free‐living birds, as reported in America and Europe over the last two decades, proved the actual viral circulation in non‐domesticated species.

Considering that studies providing a wide overview of diseases at the poultry/wild bird interface have provided valuable information in directing future research (Chen et al., 2019; Graziosi et al., 2022; Sawicka et al., 2020; Sukon et al., 2021), the present work aims to assess current knowledge on aMPV occurrence in wild birds.

### 1.2 Objectives

Given the aMPV disease burden in poultry worldwide and the evidence of a possible role played by wild birds in aMPV epidemiology, the present study summarizes aMPV serological and molecular data available in the literature on free‐ranging avifauna by conducting a systematic review and meta‐analysis. Although aMPV infection has been widely reviewed (Cook, 2000a, 2000b; Cook & Cavanagh, 2002; Jones, 1996; Kaboudi & Lachheb, 2021; Naylor & Jones, 1993; Njenga et al., 2003), to the best of our knowledge this is the first systematic review and meta‐analysis, which focuses on aMPV occurrence in wild birds.

## 2 MATERIALS AND METHODS

### 2.1 Protocol

A systematic review and metanalysis were applied to summarize data on aMPV infection in wild birds. To build our protocol, the Preferred Reporting Items for Systematic Reviews and Meta‐Analyses Protocols (PRISMA‐P) (Moher et al., 2015) and the PRISMA 2020 Statement recommendations (Page et al., 2021) were followed (Supporting Information 1).

### 2.2 Information sources and search strategy

A literature search for scientific contributions on aMPV in wild birds was conducted from 16/02/2021 to 16/12/2021. Four electronic databases were accessed including PubMed (https://pubmed.ncbi.nlm.nih.gov), the Web of Science (https://apps.webofknowledge.com/), Scopus (https://www.scopus.com/) and CAB Direct (https://www.cabdirect.org). Advanced search builders and two separate search strategies were applied, one for searching molecular studies and another for serological ones. The following keywords were used: ‘avian metapneumovirus’ or ‘avian pneumovirus’ and ‘wild birds’ or ‘free‐living birds’ together with molecular or serological method related terms (Tables 1 and 2). Filters on language (English) and timespan (studies published after 1990) were used. Manual screening of citations and reference lists of the articles retrieved were also performed to increase the chance of finding relevant publications (Higgins et al., 2021).

**TABLE 1:** Search lines used for the literature research of aMPV molecular studies in wild birds present in PubMed, Scopus, CAB Direct and Web of Science databases

Database	Search line	No. of studies retrieved
PubMed	((“pneumovirus”[Title/Abstract] OR “avian pneumovirus” OR “avian metapneumovirus ampv”[Title/Abstract] OR avian Metapneumovirus[Title/Abstract]) OR (“metapneumovirus/genetics”[MeSH Terms] OR “metapneumovirus/isolation and purification”[MeSH Terms] OR avian metapneumovirus[MeSH Terms])) AND (“animals, wild”[MeSH Terms] OR wild bird[MeSH Terms] OR (wild[Title/Abstract] AND bird*[Title/Abstract]))	28
Scopus	TITLE‐ABS ( “avian metapneumovirus” ) OR TITLE‐ABS ( “pneumovirus” ) OR TITLE‐ABS(“avian pneumovirus”) AND ( TITLE‐ABS ( “wild” ) AND TITLE‐ABS ( *bird* )) OR TITLE‐ABS ( *wild AND bird* ) AND ( TITLE‐ABS ( *detect*) OR TITLE‐ABS ( “infection” ) OR TITLE‐ABS (* isolat* ))	22
CAB Direct	(title:(Avian Pneumovirus) OR ab:(Avian Pneumovirus) OR up:(Avian Pneumovirus) OR id:(Avian Pneumovirus) OR cabicode:(Avian Pneumovirus) OR (title:(Avian metapneumovirus) OR ab:(Avian metapneumovirus) OR up:(Avian metapneumovirus) OR id:(Avian metapneumovirus) OR cabicode:(Avian metapneumovirus)) AND (title:(detection) OR ab:(detection) OR up:(detection) OR id:(detection) OR cabicode:(detection) OR title:(isolation) OR ab:(isolation) OR up:(isolation) OR id:(isolation) OR cabicode:(isolation)) AND (title:(wild bird*) OR ab:(wild bird*) OR up:(wild bird*) OR id:(wild bird*) OR cabicode:(wild bird*))	10
Web of Science	(ALL = ((“avian metapneumovirus” OR “avian pneumovirus” OR “pneumovirus”) AND (((wild) OR (free‐living)) AND (bird*)) AND (infection* OR detection* OR isolat*))	22

*Note*: Boolean operators ‘OR’ and ‘AND’ were applied. Number of scientific contributions retrieved before duplicate removal is reported.

**TABLE 2:** Search lines used for the literature research of aMPV serological studies in wild birds present in PubMed, Scopus, CAB Direct and Web of Science databases

Database	Search line	No. of studies retrieved
PubMed	((((“pneumovirus”[Title/Abstract]) OR “avian pneumovirus”[Title/Abstract]) OR “avian metapneumovirus”[Title/Abstract])) AND ((((((((Antibodies, Viral[MeSH Terms]) OR *antibody*[Title/Abstract]) OR *serolog*[Title/Abstract]) OR prevalence*[Title/Abstract]) OR Study*[Title/Abstract]) OR Survey*[Title/Abstract]) OR Survey[MeSH Terms])) AND (“animals, wild”[MeSH Terms] OR wild bird*[MeSH Terms] OR (wild[Title/Abstract] AND bird*[Title/Abstract]))	16
Scopus	TITLE‐ABS(“avian metapneumovirus”) OR TITLE‐ABS(“pneumovirus”) OR TITLE‐ABS(“avian pneumovirus”) AND (TITLE‐ABS(“wild”) AND TITLE‐ABS(*bird*)) OR TITLE‐ABS (*wild AND bird*) AND (TITLE‐ABS (*sero*) OR TITLE‐ABS (*survey*) OR TITLE‐ABS (* antibod*))	14
CAB Direct	(title:(Avian Pneumovirus) OR ab:(Avian Pneumovirus) OR up:(Avian Pneumovirus) OR id:(Avian Pneumovirus) OR cabicode:(Avian Pneumovirus) OR (title:(Avian metapneumovirus) OR ab:(Avian metapneumovirus) OR up:(Avian metapneumovirus) OR id:(Avian metapneumovirus) OR cabicode:(Avian metapneumovirus)) AND (title:(sero*) OR ab:(sero*) OR up:(sero*) OR id:(sero*) OR cabicode:(sero*) OR title:(prevalence) OR ab:(prevalence) OR up:(prevalence) OR id:(prevalence) OR cabicode:(prevalence)) AND (title:(wild bird*) OR ab:(wild bird*) OR up:(wild bird*) OR id:(wild bird*) OR cabicode:(wild bird*))	11
Web of Science	ALL = ((“avian metapneumovirus” OR “avian pneumovirus” OR “pneumovirus”) AND (((wild) OR (free‐living)) AND (bird*)) AND (antibod* OR sero* OR survey*))	14

*Note*: Boolean operators ‘OR’ and ‘AND’ were applied. Number of scientific contributions retrieved before duplicate removal is reported.

### 2.3 Selection criteria

Literature was screened by two independent investigators (G.G. and C.L.). Considering the paucity of information on aMPV occurrence in wild birds, different types of scientific contributions were considered, such as original articles, book chapters, scientific correspondence, conference proceedings, conference contributions and short communications. After duplicate removal, titles and abstracts were screened to exclude non‐relevant articles with respect to our research question (i.e. studies regarding experimental trials, studies on poultry or on intensive‐reared ducks, studies on farmed wild species). The full texts of the articles which passed the initial screening were downloaded and independently assessed for eligibility, data analysis and extraction by G.G. and C.L. If disagreements occurred, a third experienced author in the avian pathology field was consulted (E.C.). In particular, an article was considered eligible if the following requirements were met: 1) the study reported information on the occurrence of aMPV in wild bird species both free‐living or captivity kept (i.e. from wildlife rescue centres); 2) the population tested was included in two studies; 3) the species of bird tested was not identified; 4) the aMPV sero‐prevalence or viro‐prevalence outcome was not reported. If pre‐established criteria were partially met, partial data consistent with our standards were considered. Whenever the same population was surveyed in more than one publication, we would consider the contribution with the most exhaustive information. Reasons for exclusion of any study were appropriately recorded and discussed. Whenever necessary, additional information on the survey was directly sought from the respective authors.

### 2.4 Data managing and pre‐processing

The following information was extracted from each study and included in a data extraction sheet (Microsoft Excel 2021, version 16.49): first author, year of the publication, title, country, region, sampling period, taxonomic order of the host, taxonomic genus of the birds, species, age classes and sex, number of birds sampled, number of birds testing positive and diagnostic method applied. All the species were also categorized as ‘migrant’, ‘migrant/resident’ and ‘resident’ according to the region of study and the information provided by distribution maps on BirdLife International (http://datazone.birdlife.org/species/factsheet). Whenever prevalence data were expressed as percentages, raw numbers were obtained converting the percentages to the closest integers. As taxonomic inconsistencies were found, common names and species were adapted to current standards following an international online database (Gill et al., 2021). To facilitate data analysis, countries of each study were grouped into continents.

### 2.5 Study risk of bias and quality assessment

Despite current tools able to evaluate publication bias, conventional funnel plots are believed to be inaccurate for meta‐analyses of proportion with low outcomes (Hunter et al., 2014). G.G. and C.L. independently assessed the quality of the eligible studies applying the Joanna Briggs Institute (JBI) critical appraisal checklist for prevalence studies (https://jbi.global/critical‐appraisal‐tools). If the JBI checklist's outcome resulted in ‘seek for further info’, the corresponding author of a given study was contacted. Although the JBI checklist was intended for human‐related studies, it perceivably suited the data set of the study hereby presented.

### 2.6 Statistical analysis

Collected data were analysed using R software, version 4.0.4. (R Core Team, 2019) using the *meta* package. Primary outcomes of interest were the estimated overall molecular prevalence and the serological prevalence of aMPV in wild birds, calculated using a double‐arcsine transformation of data and a random‐effects model (Wang, 2018). For the pooled estimates, the Cochran's Q and the inconsistency index (*I* ^2^) were used to estimate the between‐study heterogeneity. The *I* ^2^ statistic was interpreted as small, medium or high according to <25%, 25–50% and >75% values, respectively (Higgins & Thompson, 2002). Subgroup analysis was planned to explore the potential sources of heterogeneity including the following variables: continent where the study was conducted; migration pattern of the species according to the region of the study; taxonomy of the birds (order and genus). Two independent meta‐analysis were performed, one for serological studies and another for molecular ones. Eventually, given the paucity of data for serological surveys, we performed the moderator analysis on molecular surveys only.

## 3 RESULTS

### 3.1 Literature searches

**FIGURE 1:**
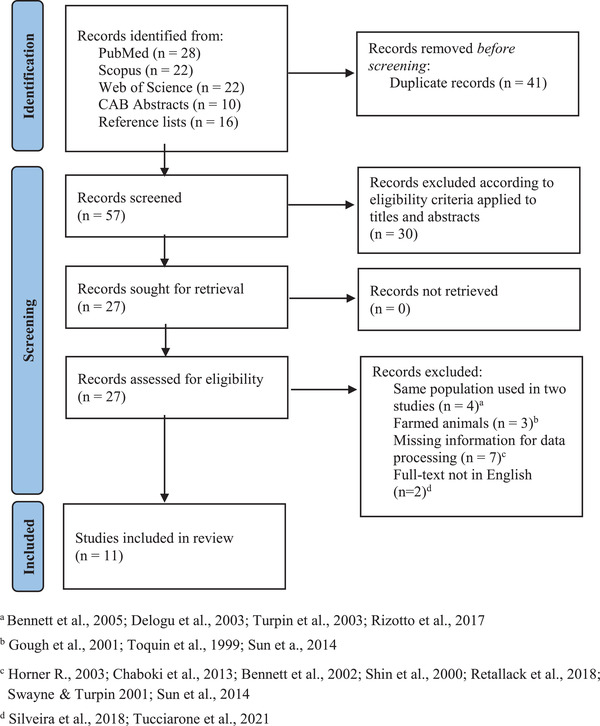
Flow diagram of the selection process of molecular studies on aMPV in free‐living wild birds, identified via databases and reference list reading

As shown in Figure 1, 98 publications on aMPV molecular surveys were retrieved through database research and reading of relevant reference lists. After duplicate records’ removal (*n* = 41), the initial screening of titles and abstracts according to pre‐established criteria brought about the exclusion of 30 more works. Altogether, 27 scientific contributions were accessed as full texts. In a secondary assessment, 16 further articles were excluded due to data already included in other publications; surveys regarding farmed wild species; prevalence data not available; preliminary results presented. Eventually, 11 publications were retained for qualitative synthesis and meta‐analysis as shown in Table 3. With regards to geographical distribution, the majority of the studies were conducted in North America (*n* = 5), followed by Europe (*n* = 4) and South America (*n* = 2). Particularly, the rank order of countries based on the number of aMPV molecular studies was United States (*n* = 3) > Canada (*n* = 2) ∼ Brazil (*n* = 2) ∼ Germany (*n* = 2) > Italy (*n* = 1) ∼ Netherlands (*n* = 1). A total of 3011 wild birds were molecularly tested for aMPV and 160 individuals tested positive.

**TABLE 3:** Details of the eligible studies on the molecular prevalence of aMPV in wild birds, sorted by continent

**Reference**	**Location**	**Period**	**Sample type**	**Taxonomic order of birds**	**Test**	**Gene targeted (subtype specificity of the test)**	**Sample size**	**Number of positives (%)**	**GenBank accession number**	**Subtype detected**
	**North America**									
Canuti et al. (2019)	Canada (Newfoundland and Labrador)	2014–2015	Oropharyngeal swab; cloacal swab	Charadriiformes; Anseriformes	Nested‐PCR on Ion Torrent sequencing products	n.a.	83	1 (1.2)	MN175553	aMPV‐*Gull Metapneumovirus* ^†^
Jardine et al. (2018)	Canada (Ontario)	2016	Oropharyngeal swab; cloacal swab	Anseriformes	qRT‐PCR	M gene (C subtype)	374	84 (22.4)	n.a.^‡^	aMPV‐C
Cha et al. (2013)	United States (n.s.^§^)	n.r.	Oropharyngeal swab	Anseriformes; Gruiformes	RT‐PCR	G genes (C subtype)	50	12 (24)	n.r.^¶^	aMPV‐C
Turpin et al. (2008)	United States (Georgia, South Carolina, Arkansas, Ohio)	2000	Choanal swab	Anseriformes; Gruiformes	RT‐PCR	M gene (A, B and C subtypes)	706	17 (2.4)	FJ195329.1‐FJ195345.1	aMPV‐C
Bennett et al. (2004)	United States (Minnesota)	2001–2002	Choanal swab; nasal turbinates tissue	Charadriiformes; Passeriformes	RT‐PCR	M gene (C subtype)	272^††^	13 (4.8)	DQ009484.1; Q2Y2M2‐Q2Y2M6	aMPV‐C
	**South America**									
Rizotto et al. (2019)	Brazil (São Paulo State)	2013–2015	Oropharyngeal swab; cloacal swab	Anseriformes; Columbiformes; Strigiformes; Falconiformes; Piciformes	RT‐PCR	N gene (A and B subtypes)	67	6 (8.9)	n.r.	aMPV‐A
Felippe et al. (2011)	Brazil (São Paulo State)	2005–2008	Tracheal swab; cloacal swab	Anseriformes; Columbiformes	RT‐PCR	G gene (A and B subtypes)	67	14 (20.9)	JF758852‐JF758858; JF758830‐JF758836	aMPV‐A; aMPV‐B
	**Europe**									
Curland et al. (2018)	Germany (Lower Saxony)	2011–2014	Tracheal sample	Galliformes	RT‐PCR	G gene (A and B subtypes)	121	8 (6.6)	n.r.	aMPV‐A or B
van Boheemen et al. (2012)	The Netherlands	n.s.	Oropharyngeal swab	Anseriformes; Charadriiformes	qRT‐PCR	L gene (A, B and C subtypes)	847	5 (0.6)	n.r.^‡‡^	aMPV‐C
Delogu et al. (2004)	Italy	2001	Choanal swab	Charadriiformes; Phenicopteriformes; Pelecaniformes	RT‐PCR	G gene (A and B subtypes)	394	0 (0.0)	n.a.	n.a.
Heffels‐Redmann et al. (1998)	Germany	1990	Conchae and tracheal sample	Charadriiformes	RT‐PCR	G gene (A and B subtypes)	30	0 (0)	n.a.	n.a.

^†^ As proposed by Canuti et al. (2019).

^‡^ n.a., not applicable.

^§^ n.s., not specified.

^¶^ n.r., not reported.

^††^Pooled samples were excluded from counts.

^‡‡^Sequences were made readily available after request to the authors.

**FIGURE 2:**
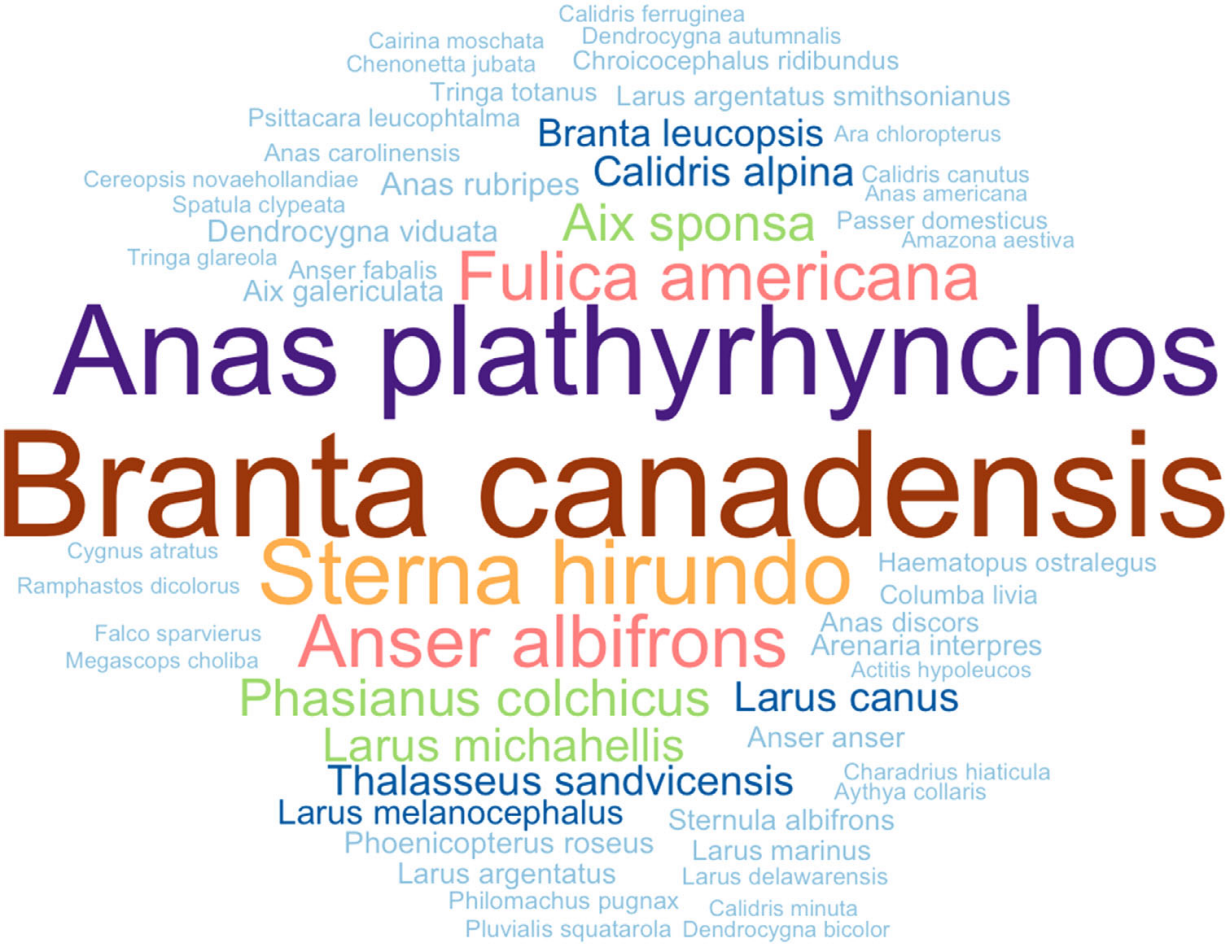
Word cloud showing the sample size of wild bird species molecularly tested for aMPV. Data are ordered from low to high, with light colours and a smaller font size for lower number of studies on a species, darker colour and bigger font size for higher number of studies

Eleven taxonomic orders of birds were surveyed: Anseriformes (*n* = 6 papers; 1598 birds tested; 101 positives), Charadriiformes (*n* = 5 papers; 951 birds tested; 8 positives); Columbiformes (*n* = 2 papers; 18 birds tested; 10 positives), Falconiformes (*n* = 1 paper; 2 birds tested; 1 positive), Galliformes (*n* = 1 paper; 121 birds tested; 8 positives), Gruiformes (*n* = 2 papers; 204 birds tested; 16 positives); Passeriformes (*n* = 1 paper; 12 birds tested; 8 positives), Phoenicopteriformes (*n* = 1 paper; 35 birds tested; 0 positive), Piciformes (*n* = 1 paper; 1 bird tested; 0 positive), Psittaciformes (*n* = 1 paper; 15 birds tested; 1 positive) and Strigiformes (*n* = 1 paper; 1 bird tested; 0 positive). Across the 11 orders above reported, 50 different species of birds belonging to 31 genera were tested; of these species, 18 resulted positive. The genera *Anas* (*n* = 3 papers) and *Larus* (*n* = 5 papers) represented the more frequently molecularly investigated for aMPV detection. The species tested are showed Figure 2. For clarification, the number of studies by order, genus and species of birds tested are summarized in Table S1 (Supporting Information 3). According to the migration patterns, *n* = 32 species were classified as ‘migrant’, *n* = 21 species as ‘resident’ and *n* = 10 species ‘migrant/resident’. With respect to the molecular method used, RT‐PCR was applied in most of the studies (*n* = 9) and qRT‐PCR in two works. The glycoprotein attachment (G) gene sequence was the most frequently targeted (Cha et al., 2013; Curland et al., 2018; Delogu et al., 2004; Felippe et al., 2011), followed by the matrix (M) gene sequence (Bennett et al., 2004; Jardine et al., 2018; Turpin et al., 2008). Lastly, the nucleoprotein (N) gene (Rizotto et al., 2019) and the large polymerase (L) gene sequences were, respectively, targeted once (van Boheemen et al., 2012), whereas Heffels‐Redmann et al. (1998) did not specify the gene used for PCR analysis. Finally, in Canuti et al. (2019) viral sequence was obtained through a PCR‐nested based genome‐walking technique on sequence fragments identified in a previous study (Verhoeven et al., 2018).

Viral isolation was attempted in five out of 11 studies through inoculation of chicken embryo fibroblast cultures (Bennett et al., 2004; Turpin et al., 2008), chicken embryonated specific pathogen free (SPF) eggs (Rizotto et al., 2019) or chicken embryo tracheal organ cultures (Heffels‐Redmann et al., 1998). In one study, virus isolation was attempted through oculonasal inoculation of SPF 3‐day‐old turkeys (Cha et al., 2013). Viral isolation was successfully achieved by Turpin et al. (2008) and Cha et al. (2013).

**FIGURE 3:**
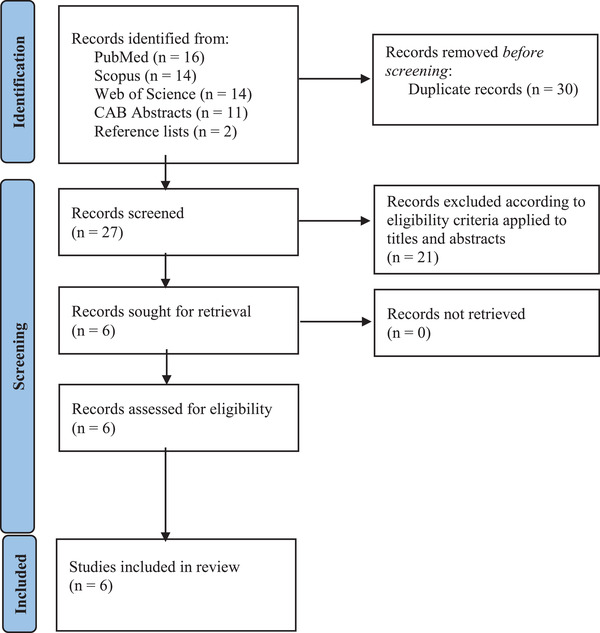
Flow diagram of the selection process of serological studies on aMPV in free‐living wild birds, identified via databases and reference list reading

**FIGURE 4:**
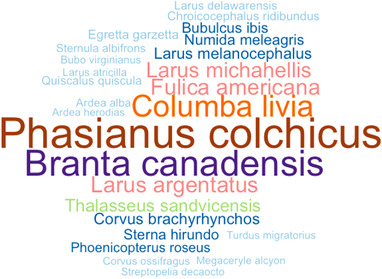
Word cloud showing the sample size of wild bird species serologically tested for aMPV. Data are ordered from low to high, with light colours and a smaller font size for lower number of studies on a species, darker colour and bigger font size for higher number of studies

As shown in Figure 3, a total of 57 contributions on aMPV serological surveys was retrieved through database research and reference list reading. After duplicate records’ removal (*n* = 30), titles and abstracts of 27 articles were screened according to the pre‐established criteria. Finally, six full texts, whose details are shown in Table 4, were included in the qualitative synthesis and meta‐analysis. Regarding geographic distribution, *n* = 2 studies were conducted in Germany, *n* = 1 in Italy, *n* = 1 in the United States and *n* = 1 in South Africa. In total, 1646 sera of wild birds were tested and 213 resulted positive. Eight different taxonomic orders were surveyed: Anseriformes (*n* = 1 paper; 310 birds tested; 103 positives), Charadriiformes (*n* = 3 papers; 421 birds tested; 45 positives), Columbiformes (*n* = 1 paper; 195 birds tested; 1 positive), Coraciiformes (*n* = 1 paper; 1 bird tested; 0 positive), Galliformes (*n* = 3 papers; 432 birds tested; 37 positives), Gruiformes (*n* = 1 paper; 114 birds tested; 20 positives), Passeriformes (*n* = 1 paper; 69 birds tested; 6 positives), Pelecaniformes (*n* = 2 papers; 69 birds tested; 1 positive), Phoenicopteriformes (*n* = 1 paper; 30 birds tested; 0 positive), Strigiformes (*n* = 1 paper; 3 birds tested; 0 positive). Across the eight orders above reported, 26 different species of birds (Figure 4) belonging to 20 genera were tested; of these species, eight resulted positive. For clarification, the number of studies by order, genus and species of birds tested are summarized in Table S2 (Supporting Information 3). *Larus* (*n* = 6 papers) was the genus most frequently tested for aMPV. According to migration patterns, *n* = 10 species surveyed across the papers were categorized as ‘migrant’, *n* = 14 species as ‘resident’ and *n* = 5 species as ‘migrant/resident’. With respect to the serological method applied, *n* = 4 studies used the enzyme‐linked immunosorbent assay (ELISA) protocol, either in house or commercial kits, whereas *n* = 2 studies used virus neutralization tests (VNT).

**TABLE 4:** Details of the eligible studies on the serological prevalence of aMPV in wild birds, sorted by continent

**Reference**	**Location**	**Period**	**Taxonomic order of birds**	**Test**	**Antigen subtype used in the method**	**Sample size**	**Number of positives (%)**
	**North America**						
Turpin et al. (2008)	United States (Georgia, South Carolina, Arkansas, Ohio)	2000–2001	Anseriformes; Charadriiformes; Columbiformes; Coraciiformes; Pelecaniformes; Passeriformes; Strigiformes; Gruiformes	ELISA^†^	Subtype C – North American lineage	732	131 (17.9)
	**Africa**						
Ratcliffe (2000)	South Africa	1997–2000	Galliformes	ELISA^‡^	n.a.^§^	17	7 (41.2)
	**Europe**						
Gethöffer et al. (2021)	Germany	2011–2015	Galliformes	VNT^¶^	n.a.	152	21 (13.8)
Delogu et al. (2004)	Italy	2001	Charadriiformes; Phenicopteriformes; Pelecaniformes	ELISA^††^	Subtype B	368	0
Catelli et al. (2001)	Italy	1992–1994	Galliformes	ELISA^††^	Subtype B	263	9 (3.4)
Heffels‐Redmann et al. (1998)	Germany	1990	Charadriiformes	VNT	Subtype A	114	45 (39.5)

^†^ In‐house enzyme‐linked immunosorbent assay (ELISA) test.

^‡^ Commercial ELISA kit (Pathasure, Cambridge Veterinary Sciences Ltd., Ely, UK).

^§^ n.a., not available.

^¶^VNT, virus neutralization test.

^††^ Commercial ELISA kit (Svanovir Avian Pneumovirus‐Ab EIA Test, SVANOVA, Biotech, Uppsala, Sweden).

### 3.2 Quality assessment

According to the JBI critical appraisal checklist for prevalence studies, all the contributions met the required standard.

### 3.3 Statistical analyses

**FIGURE 5:**
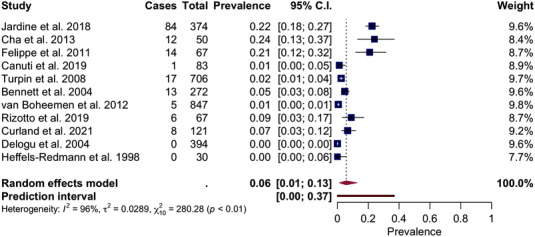
Forest plot of the random‐effects meta‐analysis of aMPV molecular prevalence. *I* ^2^ (inverse variance index), τ^2^ = the between‐study variance, χ^2^ and *p* value of the Cochran's Q test for heterogeneity

The pooled molecular prevalence of aMPV in wild birds was 6% (95% CI: 1–13%) (*I* ^2^ = 96%, *p* < .01) (Figure 5). Subgroup analyses were performed according to geographical area, taxonomic order and genus of the birds surveyed, and migration patterns (Supporting Information 2). Results suggest a significant difference between the pooled effect estimates for each geographic subgroup with the highest prevalence for South America (P: 14%, 95% CI: 1–39%), followed by North America (P: 8%, 95% CI: 2–20%) and Europe (P: 8%, 95% CI: 0–4%). Taxonomic orders and genera both appear to influence the effect estimates (*p* < .0001 each, respectively). Regarding the orders, the highest viroprevalence was detected in the Passeriformes order (P: 66%, 95% CI: 38–91%), followed by the Columbiformes order (P: 55%, 95% CI: 30–80%) and the Falconiformes order (P: 50%, 95% CI: 0–100%). According to genera, *Passer* showed the highest prevalence (P: 66%, 95% CI: 37–91%), followed by *Columba* (P: 56%, 95% CI: 30–80%) and *Falco* (P: 50%, 95% CI: 0–100%). Finally, migration patterns of the species surveyed did not appear to be significant (*p* = .1234).

**FIGURE 6:**
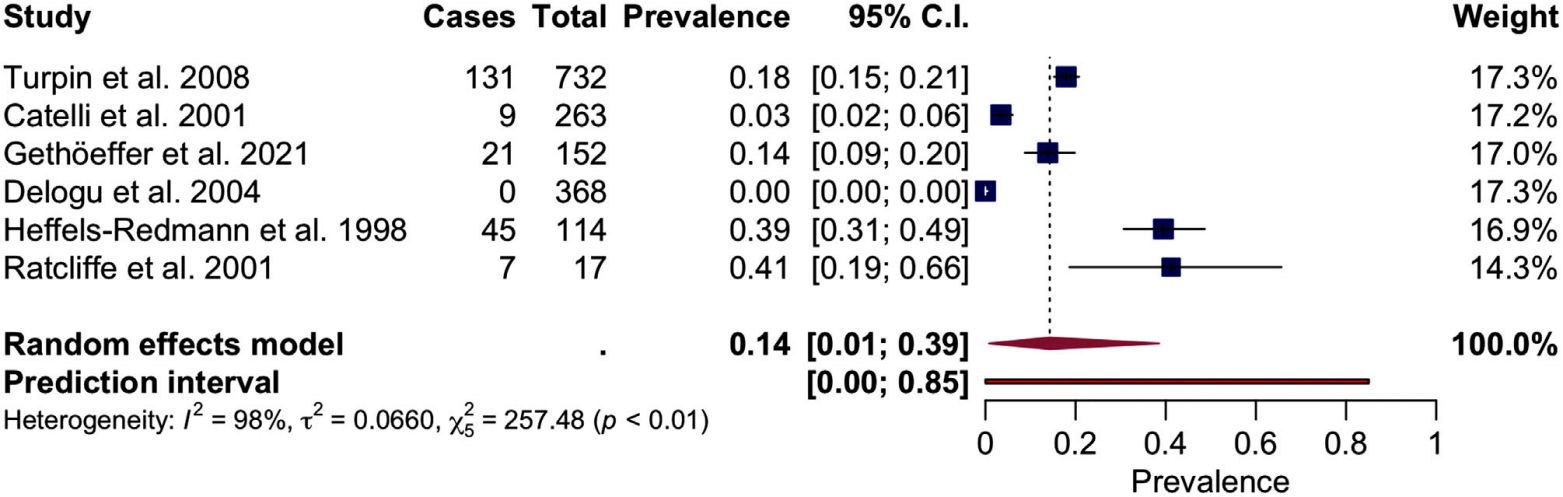
Forest plot of the random‐effects meta‐analysis of aMPV serological prevalence. *I* ^2^ (inverse variance index), τ^2^ = the between‐study variance, χ^2^ and *p* value of the Cochran's Q test for heterogeneity

With respect to the pooled seroprevalence of aMPV in wild birds, it was estimated as 14% (95% CI: 1–39%) (*I* ^2^ = 98%, *p* < .01; Figure 6). Given the lack of data available, subgroup analyses according to geographical area, taxonomic order of birds surveyed, genera and migration patterns were not performed for serological studies.

## 4 DISCUSSION

### 4.1 Summary of evidence

The potential role of wild birds as reservoir hosts or epiphenomena with respect to aMPV epidemiology has not been fully disclosed yet. Despite limited data available, a systematic revision of literature and meta‐analysis were, for the first time, applied in our study to the abovementioned question to summarize the sero–viroprevalence of aMPV in wild birds, as a necessary step to identify research gaps and encourage future study directions.

Literature search strategies hereby applied retrieved more molecular studies than serological ones, probably due to the increase in the use of PCR‐based technologies with respect to serology for the detection of infectious agents. The overall results of the meta‐analyses showed a moderate exposure of wild birds to aMPV (aMPV molecular prevalence: 6%; aMPV serological prevalence: 14%). Given the high degree of between‐study heterogeneity evidenced by the meta‐analyses conducted, the pooled estimates need to be considered together with the 95% CI.

Possible sources of heterogeneity may be related to the different sensitivities and specificities of the assays used, the area studied, the different sample type analysed, and the different ecology and habits of the wild birds sampled.

The targeting of different gene segments in molecular studies likely influenced the results obtained when testing wild birds, due to the different subtype specificity of the RT‐PCRs assays used (Jones & Rautenschlein, 2013). With respect to serological surveys, the use of tests having different sensitivity and specificity levels could have further influenced the accuracy of the results obtained.

According to subgroup analyses, our results showed a statistically significant association between positive aMPV molecular findings and the location of the samplings (*p* = .01). However, considering that only three categorical variables were extracted from our data set with respect to the geographical distribution of the studies (North America, South America and Europe), we believe this to be poorly representative of global trends. With respect to North America, initial aMPV detections were linked to spillover events from poultry to free‐living wild species. The first aMPV‐C molecular detections (Shin et al., 2000) and successful viral isolation in a healthy live‐captured Canada goose (*B. canadensis*) in Minnesota (Bennett et al., 2002) led the authors to consider wild birds as natural viral reservoirs. Subsequently, aMPV‐C occurrence outside poultry USA endemic territories was reported by Turpin et al. (2008), who detected both antibodies and viral RNA of aMPV‐C in American coots (*Fulica americana* gmelin, 1789) and in Canada geese (*B. canadensis*) in Georgia, South Carolina, Arkansas and Ohio. aMPV‐C direct evidence in wild species was also identified in aquatic birds by Jardine et al. (2018) in Canada where aMPV infection has never been reported for poultry. Considering South America, aMPV subtypes A and B were detected in numerous free‐living wild species and in individuals hosted in rehabilitation centres (Felippe et al., 2011; Rizotto et al., 2019), probably implying aMPV spread between the birds sampled in captivity. Concerning Europe, viral subtype C was molecularly detected in wild mallards (*A. platyrhynchos*), graylag geese (*Anser anser* (linnaeus, 1758)) and common gulls (*Larus canus* linnaeus, 1758) sampled in the Netherlands (van Boheemen et al., 2012) and in wild anatids sampled in Italy (Graziosi et al., 2022; Tucciarone et al., 2022). aMPV was also detected by RT‐PCR in free‐living pheasants (*P. colchicus*) in Germany (Curland et al., 2018), without further viral subtyping.

Statistical comparison among orders and genera of molecularly tested wild birds revealed a significant association (*p* < .0001) between these moderators and the aMPV molecular prevalence. Nevertheless, not all the taxa included in the subgroup analysis were represented in more than one study. The orders Passeriformes, Columbiformes and Falconiformes showed the highest molecular/viroprevalence outcomes, although sampling bias due to under‐sampling probably influenced the results. In fact, the Passeriformes order, represented solely by the house sparrow (*Passer domesticus* (linnaeus, 1758)), and the Falconiformes order, represented by the American kestrel (*Falco sparvierus* linnaeus, 1758), were both sampled once (Bennett et al., 2004; Rizotto et al., 2019). Considering Columbiformes, this order also included a single species sampled, the pigeon (*Columba livia* GMELIN, 1789), which was surveyed twice (Felippe et al., 2011; Rizotto et al., 2019). House sparrows, American kestrels and pigeons are common rural species inhabiting agroecosystems, which could therefore encounter aMPV by frequenting poultry farm surroundings. Regarding house sparrows and/or pigeons, results of experimental infection with aMPV are controversial (Catelli et al., 2012; Gharaibeh & Shamoun, 2012; Gough et al., 1988). Current evidence does not allow a clear epidemiological role to be assigned to these species, although they appear more likely to be epiphenomena.

Taken as a whole, molecular and serological evidence suggests that some wild bird taxa could play a role in aMPV epidemiology. Particularly, wild ducks, geese, pheasants and gulls, according to scientific contributions hereby considered, proved to be susceptible to aMPV infection. Considering the Eurasian lineage of aMPV‐C as well adapted to ducks (Brown et al., 2019), further investigation on wild anatids may lead to disclosure of potential aMPV reservoir species. Although moderator analysis on the association between aMPV molecular detection and migration patterns resulted in a non‐statistical significance, migratory or non‐migratory attitudes of species still deserve to be considered for further consideration. It can be fairly hypothesized that migratory birds may condition the spread of aMPV along migratory flight paths, especially for the aMPV subtype C, as already proven for avian influenza (Global Consortium for H5N8 and Related Influenza Viruses, 2016). In particular, the detection of aMPV‐C in wild migratory species sampled in territories where this subtype has never been reported for poultry (van Boheemen et al., 2012; Graziosi et al., 2022; Jardine et al., 2018; Turpin et al., 2008) corroborates this hypothesis. Separate migratory attitudes of Eurasian and American wildfowl could also be relevant in shaping aMPV‐C genetic diversity, which already recognizes two distinct lineages.

On the other hand, with respect to resident wild bird species, these could possibly play a bridging role in between‐poultry farm or between‐potential natural reservoir species and poultry. Considering aMPV‐A, B or C (North American lineage) as more adapted to gallinaceous birds (Brown et al., 2019), wild Galliformes such as pheasants could therefore be involved in the viral epidemiology. Evidence of aMPV‐A or B subtype infection in free‐living or farmed pheasants (Catelli et al., 2001; Curland et al., 2018; Gough et al., 2001; Welchman et al., 2002), and the molecular detection of aMPV‐C in pheasants sampled in a live‐bird market (Lee et al., 2007) in South Korea, enlighten the potential impact of this territorial species with respect to local aMPV circulation at the poultry/wildlife interface with free‐range pheasants being frequently observed in rural areas or around poultry farms.

Finally, with respect to gulls, their trophic plasticity and the evidence of their susceptibility to aMPV (Canuti et al., 2019; Heffels‐Redmann et al., 1998; van Boheemen et al., 2012) suggest their possible involvement in viral epidemiology. *Larus* are indeed regarded as generalist carnivores, which use different habitats as nesting sites and consume both marine and terrestrial food resources (Belant et al., 1998; Shaffer et al., 2017).

### 4.2 Limitations

Among the constraints identified regarding the systematic review and meta‐analysis hereby presented, we acknowledge the scarcity of publications on aMPV occurrence in free‐ranging birds in comparison with the abundance of studies available for poultry. In that sense, a possible limitation of our study might be due to a lack of data from Africa and Asiatic countries, which may generate a misinterpretation of the actual geographic distribution and prevalence of known aMPV subtypes in wild birds.

We also suggest the existence of possible research which may have not been accessible through the search strategy adopted. With respect to the statistical analysis, the scarcity of eligible articles concerning aMPV serological surveys prevented us from performing subgroup analyses and exploring the sources of the between‐study heterogeneity recorded. Moreover, outlier data were not statistically identified to avoid further decreasing of the overall number of studies considered.

Finally, any statistical test to quantify publication bias was not applied due to the absence of specific tools applicable to studies on proportions (Murad et al., 2018; Olsen et al., 2019).

## 5 CONCLUSION

Further aMPV surveys in wild birds are encouraged for a better comprehension of aMPV epidemiology and to better characterize the virus host breadth. Considering aMPV as transmitted by direct contact, the gregariousness of wild species could be an important trait to be considered in selecting the species to be tested. Furthermore, accurate recording of taxonomic and demographic information of individuals tested, especially sex and age classes, would be essential to notice specific aMPV infection patterns. Moreover, sampling location data would support the understanding of epidemiological links between wild free‐ranging avifauna and aMPV occurrence on poultry farms. Whenever positive results are discussed, a thorough examination of the host ecology may allow further epidemiological considerations. With respect to the diagnostic methods applied, in order to avoid underestimation of the circulation of aMPV in new bird taxa and reveal unobvious aspects of viral distribution in wild hosts, it is strongly recommended to choose molecular protocols that are not subtype‐specific and to proceed with further characterization of positive findings by sequencing.

## AUTHOR CONTRIBUTIONS

Conceptualization: G.G, C.L. and E.C. Acquisition of data: G.G. and C.L. Statistical analysis: G.G. Interpretation of data: G.G. and C.L. Writing – original draft preparation: G.G. Writing – review and editing: G.G., E.C. and C.L. Supervision: E.C. and C.L. All authors have read and agreed to the published version of the manuscript.

## CONFLICT OF INTEREST

The authors declare that they have no competing interests.

## ETHICAL APPROVAL STATEMENT

Not required as this is a systematic review.

## Supporting information



Supporting Material

Supporting Material

Supporting Material

## Data Availability

The data that support the findings of this study are available in the supplementary material of this article.
