# Supplementary material for: Disentangling the role of wild birds in avian metapneumovirus (aMPV) epidemiology: A systematic review and meta‐analysis
Source: Transbound Emerg Dis. 2022 Aug 22;69(6):3285–99. doi: 10.1111/tbed.14680 (PMC10086952; doi:10.1111/tbed.14680)
Supplement: Supplementary file 1 — Supporting Material [file TBED-69-3285-s003.docx]

**Supporting Information 1: Systematic review and Meta-Analysis Protocol (PRISMA-P)**

**ADMINISTRATIVE INFORMATION**

**Title:**

Serological and molecular prevalence of Avian metapneumovirus in free-living wild birds on a global scale: protocol for a systematic review

**Authors**

Giulia Graziosi: [giulia.graziosi2@unibo.it](mailto:giulia.graziosi2@unibo.it)

Caterina Lupini: [caterina.lupini@unibo.it](mailto:caterina.lupini@unibo.it)

Elena Catelli: [elena.catelli@unibo.it](mailto:elena.catelli@unibo.it)

**Contributions**

EC and CL conceptualized the research question. GG and CL perform the literature search, collect and extract data from eligible studies. EC double checks the quality of data extraction. GG analyses and interprets the data.

**Funding sources/sponsors**

No external funding sought

**INTRODUCTION**

**Rationale**

Avian metapneumovirus (aMPV) infection is considered as an economical and welfare issue to the global poultry industry. Whereas aMPV infection is well known in poultry, wild free-living birds are believed to be susceptible with an unclear epidemiological role. We carry out a systematic review and meta-analysis to assess the sero-prevalence and the viral prevalence of aMPV in wild free-living birds on a global scale.

**Objectives**

To assess the seroprevalence and molecular prevalence of aMPV in wild free-living wild birds. Our systematic review may help to direct future epidemiological surveys of aMPV in wild avian hosts and to enlighten the potential epidemiological role of defined species.

**METHODS**

**Eligibility criteria**

All studies (journal articles, conference abstracts, conference proceedings, theses, reports, scientific article manuscripts) on aMPV (sero-surveys and viro-surveys) in wild free-living birds published after 1970. Studies are excluded if the sample size ≤10 and if the same population is used in two studies.

**Information sources**

Iterative searches are carried out of the following repositories: PubMed, CAB Abstracts, Scopus and Web of Sciences.

Dates of coverage: January 2021 – September 2021

**Search strategy**

Two different search strategies were applied with respect to molecular (1A) and serological (1B) studies on aMPV in wild birds.

1A. Molecular studies on aMPV search lines.

| Database | Search line | No. of studies retrieved |
| --- | --- | --- |
| PubMed | (("pneumovirus"[Title/Abstract] OR “avian pneumovirus” OR "avian metapneumovirus ampv"[Title/Abstract] OR avian Metapneumovirus[Title/Abstract]) OR ("metapneumovirus/genetics"[MeSH Terms] OR "metapneumovirus/isolation and purification"[MeSH Terms] OR avian metapneumovirus[MeSH Terms])) AND (“animals, wild”[MeSH Terms] OR wild bird[MeSH Terms] OR (wild[Title/Abstract] AND bird*[Title/Abstract])) | 28 |
| Scopus | TITLE-ABS ( "avian metapneumovirus" )  OR  TITLE-ABS ( "pneumovirus" ) OR TITLE-ABS(“avian pneumovirus”)  AND  ( TITLE-ABS ( "wild" )  AND  TITLE-ABS ( *bird* ))  OR  TITLE-ABS ( *wild  AND bird* )  AND  ( TITLE-ABS ( *detect*)  OR  TITLE-ABS ( "infection" )  OR  TITLE-ABS (* isolat* )) | 22 |
| CAB  Direct | (title:(Avian Pneumovirus) OR ab:(Avian Pneumovirus) OR up:(Avian Pneumovirus) OR id:(Avian Pneumovirus) OR cabicode:(Avian Pneumovirus) OR (title:(Avian metapneumovirus) OR ab:( Avian metapneumovirus) OR up:( Avian metapneumovirus) OR id:( Avian metapneumovirus) OR cabicode:( Avian metapneumovirus)) AND (title:(detection) OR ab:(detection) OR up:(detection) OR id:(detection) OR cabicode:(detection) OR title:(isolation) OR ab:(isolation) OR up:(isolation) OR id:(isolation) OR cabicode:(isolation)) AND (title:(wild bird*) OR ab:(wild bird*) OR up:(wild bird*) OR id:(wild bird*) OR cabicode:(wild bird*)) | 10 |
| Web of Science | (ALL=(("avian metapneumovirus" OR "avian pneumovirus" OR “pneumovirus”) AND (((wild) OR (free-living)) AND (bird*)) AND (infection* OR detection* OR isolat*)) | 22 |

1B. Serological studies on aMPV search lines.

| Database | Search line | No. of studies retrieved |
| --- | --- | --- |
| PubMed | (((("pneumovirus"[Title/Abstract]) OR “avian pneumovirus”[Title/Abstract]) OR "avian metapneumovirus"[Title/Abstract])) AND ((((((((Antibodies, Viral[MeSH Terms]) OR *antibody*[Title/Abstract]) OR *serolog*[Title/Abstract]) OR prevalence*[Title/Abstract]) OR Study*[Title/Abstract]) OR Survey*[Title/Abstract]) OR Survey[MeSH Terms])) AND (“animals, wild”[MeSH Terms] OR wild bird*[MeSH Terms] OR (wild[Title/Abstract] AND bird*[Title/Abstract])) | 16 |
| Scopus | TITLE-ABS("avian metapneumovirus") OR TITLE-ABS("pneumovirus") OR TITLE-ABS(“avian pneumovirus”) AND (TITLE-ABS("wild") AND TITLE-ABS(*bird* )) OR TITLE-ABS ( *wild AND bird* ) AND ( TITLE-ABS ( *sero*) OR TITLE-ABS ( *survey* ) OR TITLE-ABS (* antibod* )) | 14 |
| CAB  Direct | (title:(Avian Pneumovirus) OR ab:(Avian Pneumovirus) OR up:(Avian Pneumovirus) OR id:(Avian Pneumovirus) OR cabicode:(Avian Pneumovirus) OR (title:(Avian metapneumovirus) OR ab:( Avian metapneumovirus) OR up:( Avian metapneumovirus) OR id:( Avian metapneumovirus) OR cabicode:( Avian metapneumovirus)) AND (title:(sero*) OR ab:(sero*) OR up:(sero*) OR id:(sero*) OR cabicode:(sero*) OR title:(prevalence) OR ab:(prevalence) OR up:(prevalence) OR id:(prevalence) OR cabicode:(prevalence)) AND (title:(wild bird*) OR ab:(wild bird*) OR up:(wild bird*) OR id:(wild bird*) OR cabicode:(wild bird*)) | 11 |
| Web of Science | ALL=(("avian metapneumovirus" OR "avian pneumovirus" OR “pneumovirus”) AND (((wild) OR (free-living)) AND (bird*)) AND (antibod* OR sero* OR survey*)) | 14 |

**Study records**

**Data management, selection process and data collection process**

Data extraction was performed by two independent reviewers and the quality of the data was double checked by a third author. Whenever two different diagnostic methods were applied, only the outcomes from the test used as confirmatory were considered. If data retrieved from original articles were expressed as percentages, raw number were obtained converting the percentages to the closer integer.

Two data extraction sheets were created on Microsoft Excel 2021 (version 16.49): 1) for the molecular prevalence and 2) for the seroprevalence.

The first was filled with the following information: title, first author, year of publication, country, sampling period, host identification (order, family, and species), age classes, total number of animals tested, total number of positive cases, type of sample collected, molecular method, specificity and sensitivity of the method applied, molecular method, subtype characterization (A, B, C, D), GenBank accession number (<http://www.ncbi.nlm.nih.gov/>) and other relevant comments.

The second with: title, first author, year of publication, country, sampling period, host identification (order, family and species), age classes, total number of animals tested, total number of positive cases, serological technique, specificity and sensitivity of the method applied, serological cut-off to define a sample as positive, subtype detected (A, B, C, D) and other relevant comments.

**Data items – RNA prevalence**

| **Variable** | **Explanation** |
| --- | --- |
| **Title** | Write the complete title |
| **First author** | Write the last name of the first author |
| **Year of publication** | Write the year when the study was published in the journal or take the date from the respective source (i.e. conference abstracts) |
| **Country** | Write the country where samples were collected |
| **Sampling period** | Write the years when the samples started and finished to be collected |
| **Host identification** | Write the taxonomic Order, Family and the species of the host |
| **Age classes** | Write the age classes of the host |
| **Type of sample** | Write the type of sample analyzed (e.g. oropharyngeal swab, choanal clefts) |
| **Total number of animals (N)** | Write the total number of animals tested in the study |
| **Total number of cases (n)** | Write the total number of animals that were aMPV positive |
| **Molecular technique** | Write the type of the molecular technique used |
| **Se (%)** | Write test sensitivity |
| **Sp (%)** | Write test specificity |
| **Subtype** | Write the aMPV subtype (A, B, C, D) |
| **GenBank accession number** | Write GenBank accession number |
| **Other comments** | Write any comment relevant to interpret the RNA prevalence |

If an object is not provided, ‘N.R.’ (NOT REPORTED) is written. If some data are difficult to extract, a comment is written in that cell.

**Data items – seroprevalence**

| **Variable** | **Explanation** |
| --- | --- |
| **Title** | Write the complete title |
| **First author** | Write the last name of the first author |
| **Year of publication** | Write the year when the study was published in the journal or take the date from the respective source (i.e. conference abstracts) |
| **Country** | Write the country where samples were collected |
| **Sampling period** | Write the years during which the samples were collected |
| **Host identification** | Write the taxonomic Order, Family and the species of the host |
| **Age classes** | Write the age classes of the host |
| **Total number of animals (N)** | Write the total number of animals tested in the study |
| **Total number of cases (n)** | Write the total number of seropositive animals |
| **Serological method** | Write the type of the serologic test used |
| **Serological test details** | Write if in-house test or specify test trade name (if commercial test) |
| **Cut-off value used** | Write the cut-off to identify a positive case |
| **Se (%)** | Write test sensitivity |
| **Sp (%)** | Write test specificity |
| **Subtype** | Write the aMPV subtype (A, B, C, D) |
| **Other comments** | Write any comment relevant to interpret the seroprevalence |

If the object is not provided, ‘N.R.’ (NOT REPORTED) is written. If some data are difficult to extract, a comment is written in that cell.

**Primary outcomes**

Estimate of the RNA-prevalence and the sero-prevalence of aMPV in wild free-living birds without any geographic restriction (positive cases / total number of animals tested).

**Additional outcomes**

Qualitative analyses of categorical variables related to the wild hosts as possibly relevant to further epidemiological considerations (taxonomic order of the host, taxonomic genus, country, migration pattern).

**Risk of bias in individual studies**

To minimize the risk of bias in individual studies, anything that could potentially affect the interpretation of the seroprevalence will be written in the comment section of data extraction. Furthermore, the JBI Critical Appraisal Checklist for studies reporting prevalence data will be used to assess the quality of the studies.

**Data synthesis**

If studies are sufficiently homogeneous, a meta-analyses using a random-effects model is carried out and the inverse variance index I^2^ is used to quantify heterogeneity as low (I^2^ <25%), moderate (I^2^: 25%-75%) and high (I^2^ >75%) heterogeneity.

Subgroup analyses according to geographic area, taxonomic order, genus of the birds and phenology (migrant/resident) could be performed if data are consistent.

**PRISMA-P (Preferred Reporting Items for Systematic review and Meta-Analysis Protocols) 2020 checklist: recommended items to address in a systematic review protocol**

| **Section and Topic** | **Item #** | **Checklist item** | **Location where item is reported** |
| --- | --- | --- | --- |
| **TITLE** | | |  |
| Title | 1 | Identify the report as a systematic review. | p.1 |
| **ABSTRACT** | | |  |
| Abstract | 2 | See the PRISMA 2020 for Abstracts checklist. | p.1 |
| **INTRODUCTION** | | |  |
| Rationale | 3 | Describe the rationale for the review in the context of existing knowledge. | p.1 |
| Objectives | 4 | Provide an explicit statement of the objective(s) or question(s) the review addresses. | p.2 |
| **METHODS** | | |  |
| Eligibility criteria | 5 | Specify the inclusion and exclusion criteria for the review and how studies were grouped for the syntheses. | p.3 |
| Information sources | 6 | Specify all databases, registers, websites, organisations, reference lists and other sources searched or consulted to identify studies. Specify the date when each source was last searched or consulted. | p.3 |
| Search strategy | 7 | Present the full search strategies for all databases, registers and websites, including any filters and limits used. | Table 1 and Table 2 |
| Selection process | 8 | Specify the methods used to decide whether a study met the inclusion criteria of the review, including how many reviewers screened each record and each report retrieved, whether they worked independently, and if applicable, details of automation tools used in the process. | p.3 |
| Data collection process | 9 | Specify the methods used to collect data from reports, including how many reviewers collected data from each report, whether they worked independently, any processes for obtaining or confirming data from study investigators, and if applicable, details of automation tools used in the process. | p.3 |
| Data items | 10a | List and define all outcomes for which data were sought. Specify whether all results that were compatible with each outcome domain in each study were sought (e.g. for all measures, time points, analyses), and if not, the methods used to decide which results to collect. | p.3 |
|  | 10b | List and define all other variables for which data were sought (e.g. participant and intervention characteristics, funding sources). Describe any assumptions made about any missing or unclear information. | p.3 |
| Study risk of bias assessment | 11 | Specify the methods used to assess risk of bias in the included studies, including details of the tool(s) used, how many reviewers assessed each study and whether they worked independently, and if applicable, details of automation tools used in the process. | p.3 |
| Effect measures | 12 | Specify for each outcome the effect measure(s) (e.g. risk ratio, mean difference) used in the synthesis or presentation of results. | p.3 |
| Synthesis methods | 13a | Describe the processes used to decide which studies were eligible for each synthesis (e.g. tabulating the study intervention characteristics and comparing against the planned groups for each synthesis (item #5)). | p.3 |
|  | 13b | Describe any methods required to prepare the data for presentation or synthesis, such as handling of missing summary statistics, or data conversions. | p.3 |
|  | 13c | Describe any methods used to tabulate or visually display results of individual studies and syntheses. | p.3 |
|  | 13d | Describe any methods used to synthesize results and provide a rationale for the choice(s). If meta-analysis was performed, describe the model(s), method(s) to identify the presence and extent of statistical heterogeneity, and software package(s) used. | p.4 |
|  | 13e | Describe any methods used to explore possible causes of heterogeneity among study results (e.g. subgroup analysis, meta-regression). | p.4 |
|  | 13f | Describe any sensitivity analyses conducted to assess robustness of the synthesized results. | Not applicable |
| Reporting bias assessment | 14 | Describe any methods used to assess risk of bias due to missing results in a synthesis (arising from reporting biases). | Not applicable |
| Certainty assessment | 15 | Describe any methods used to assess certainty (or confidence) in the body of evidence for an outcome. | Not applicable |
| **RESULTS** | | |  |
| Study selection | 16a | Describe the results of the search and selection process, from the number of records identified in the search to the number of studies included in the review, ideally using a flow diagram. | p.4 |
|  | 16b | Cite studies that might appear to meet the inclusion criteria, but which were excluded, and explain why they were excluded. | p.4 |
| Study characteristics | 17 | Cite each included study and present its characteristics. | Table 3 and Table 4 |
| Risk of bias in studies | 18 | Present assessments of risk of bias for each included study. | p.5 |
| Results of individual studies | 19 | For all outcomes, present, for each study: (a) summary statistics for each group (where appropriate) and (b) an effect estimate and its precision (e.g. confidence/credible interval), ideally using structured tables or plots. | Table 3 and Table 4 |
| Results of syntheses | 20a | For each synthesis, briefly summarise the characteristics and risk of bias among contributing studies. | Not applicable |
|  | 20b | Present results of all statistical syntheses conducted. If meta-analysis was done, present for each the summary estimate and its precision (e.g. confidence/credible interval) and measures of statistical heterogeneity. If comparing groups, describe the direction of the effect. | p.5 |
|  | 20c | Present results of all investigations of possible causes of heterogeneity among study results. | p.5 |
|  | 20d | Present results of all sensitivity analyses conducted to assess the robustness of the synthesized results. | Not applicable |
| Reporting biases | 21 | Present assessments of risk of bias due to missing results (arising from reporting biases) for each synthesis assessed. | Not applicable |
| Certainty of evidence | 22 | Present assessments of certainty (or confidence) in the body of evidence for each outcome assessed. | Not applicable |
| **DISCUSSION** | | |  |
| Discussion | 23a | Provide a general interpretation of the results in the context of other evidence. | p.6 |
|  | 23b | Discuss any limitations of the evidence included in the review. | p.7 |
|  | 23c | Discuss any limitations of the review processes used. | p.7 |
|  | 23d | Discuss implications of the results for practice, policy, and future research. | p.8 |
| **OTHER INFORMATION** | | |  |
| Registration and protocol | 24a | Provide registration information for the review, including register name and registration number, or state that the review was not registered. | Supplementary Materials 1 |
|  | 24b | Indicate where the review protocol can be accessed, or state that a protocol was not prepared. | Supplementary Materials 1 |
|  | 24c | Describe and explain any amendments to information provided at registration or in the protocol. | Not applicable |
| Support | 25 | Describe sources of financial or non-financial support for the review, and the role of the funders or sponsors in the review. | p.8 |
| Competing interests | 26 | Declare any competing interests of review authors. | p.8 |
| Availability of data, code and other materials | 27 | Report which of the following are publicly available and where they can be found: template data collection forms; data extracted from included studies; data used for all analyses; analytic code; any other materials used in the review. | p.2; p.5 |

*From:*  Page MJ, McKenzie JE, Bossuyt PM, Boutron I, Hoffmann TC, Mulrow CD, et al. The PRISMA 2020 statement: an updated guideline for reporting systematic reviews. BMJ 2021;372:n71. doi: 10.1136/bmj.n71

For more information, visit: <http://www.prisma-statement.org/>
