# Supplementary material for: Disentangling the role of wild birds in avian metapneumovirus (aMPV) epidemiology: A systematic review and meta‐analysis
Source: Transbound Emerg Dis. 2022 Aug 22;69(6):3285–99. doi: 10.1111/tbed.14680 (PMC10086952; doi:10.1111/tbed.14680)
Supplement: Supplementary file 2 — Supporting Material [file TBED-69-3285-s001.docx]

**Supporting Information 2: Moderator analyses of aMPV molecular studies**

Moderator analyses of eligible aMPV molecular studies according to the criteria applied for the systematic revision of the literature. Sub-group analyses were independently performed considering the geographical distribution of the studies, the migration pattern of the birds sampled, the taxonomic order and the genus of birds tested for aMPV detection. Statistically significant differences between groups are reported in bold.

| **Subgroup** | **No of prevalence inputs** | **Sample size** | **Proportion** | **95% CI** | **I^2^ (%)** | **Difference between groups** |
| --- | --- | --- | --- | --- | --- | --- |
| **Geographical distribution** | | | | | | **p=0.01** |
| Northern America | 5 | 1485 | 0.08 | 0.16 – 0.20 | 97 |  |
| Southern America | 2 | 134 | 0.14 | 0.05 – 0.28 | 73.1 |  |
| Europe | 4 | 1392 | 0.08 | 0 – 0.043 | 86.2 |  |
| **Migration pattern** | | | | | | p=0.17 |
| Migrant | 32 | 1216 | 0.0 | 0 – 0.02 | 66.6 |  |
| Resident | 21 | 482 | 0.01 | 0 - 0.10 | 76.5 |  |
| Migrant/resident | 10 | 1260 | 0.08 | 0.03 - 0.20 | 95.6 |  |
| **Taxonomic order of the birds sampled** | | | | | | **p<0.0001** |
| Anseriformes | 6 | 1598 | 0.05 | 0 – 0.18 | 97.5 |  |
| Charadriiformes | 5 | 951 | 0.004 | 0 – 0.02 | 61.5 |  |
| Columbiformes | 2 | 18 | 0.56 | 0 – 1.0 | 0 |  |
| Falconiformes | 1 | 2 | 0.50 | 0 – 1.0 | -- |  |
| Galliformes | 1 | 121 | 0.06 | 0.02 – 0.11 | -- |  |
| Gruiformes | 2 | 204 | 0.11 | 0 – 0.1.0 | 79.9 |  |
| Passeriformes | 1 | 12 | 0.67 | 0.37 – 0.91 | -- |  |
| Phoenicopteriformes | 1 | 35 | 0 | 0 – 0.05 | -- |  |
| Piciformes | 1 | 1 | 0 | 0 – 1.00 | -- |  |
| Psittaciformes | 1 | 15 | 0.06 | 0 – 0.26 |  |  |
| Strigiformes | 1 | 1 | 0 | 0 – 1.00 | -- |  |
| **Genus of the birds sampled** | | | | | | **p<0.0001** |
| *Actitis* | 1 | 1 | 0 | 0 – 1.00 | -- |  |
| *Aix* | 1 | 121 | 0.01 | 0 – 0.03 | -- |  |
| *Amazona* | 1 | 1 | 0 | 0 – 1.00 | -- |  |
| *Anas* | 3 | 524 | 0.06 | 0 – 0.33 | 98.7 |  |
| *Anser* | 1 | 269 | 0 | 0 – 0.07 | -- |  |
| *Arenaria* | 1 | 30 | 0 | 0 – 0.6 | -- |  |
| *Aythya* | 1 | 6 | 0 | 0 – 0.27 | -- |  |
| *Branta* | 3 | 628 | 0.04 | 0 – 0.24 | 90.5 |  |
| *Cairina* | 1 | 1 | 0 | 0 – 1.00 | -- |  |
| *Calidris* | 2 | 106 | 0 | 0 – 0 | 0 |  |
| *Charadrius* | 1 | 5 | 0 | 0 – 0.31 | -- |  |
| *Chroicocephalus* | 1 | 18 | 0 | 0 – 0.09 | -- |  |
| *Columba* | 2 | 18 | 0.56 | 0.30 – 0.80 | 0 |  |
| *Dendrocygna* | 1 | 38 | 0.02 | 0 – 0.02 | 0 |  |
| *Falco* | 1 | 2 | 0.50 | 0 – 1.00 | -- |  |
| *Fulica* | 2 | 204 | 0.11 | 0 – 0.31 | 79.9 |  |
| *Haematopus* | 1 | 18 | 0 | 0 – 0.10 | -- |  |
| *Larus* | 5 | 279 | 0.05 | 0 – 0.14 | 85.8 |  |
| *Mareca* | 1 | 1 | 0 | 0 - 0 | -- |  |
| *Megascops* | 1 | 1 | 0 | 0 – 1.00 | -- |  |
| *Passer* | 1 | 12 | 0.66 | 0.37 – 0.91 | -- |  |
| *Phasianus* | 1 | 121 | 0.06 | 0.03 – 0.11 | -- |  |
| *Phoenicopterus* | 1 | 35 | 0 | 0 – 0.05 | -- |  |
| *Pluvialis* | 1 | 7 | 0 | 0 – 0.23 | -- |  |
| *Psittacara* | 1 | 14 | 0.07 | 0 – 0.28 | -- |  |
| *Ramphastos* | 1 | 1 | 0 | 0 – 1.00 | -- |  |
| *Spatula* | 1 | 5 | 0.4 | 0.02 – 0.86 | -- |  |
| *Sterna* | 3 | 300 | 0 | 0 - 0 | 0 |  |
| *Sternula* | 1 | 30 | 0 | 0 – 0.06 | -- |  |
| *Thalasseus* | 1 | 86 | 0 | 0 – 0.02 | -- |  |
| *Tringa* | 2 | 18 | 0 | 0 – 0.05 | 0 |  |
