# Supplementary material for: Disentangling the role of wild birds in avian metapneumovirus (aMPV) epidemiology: A systematic review and meta‐analysis
Source: Transbound Emerg Dis. 2022 Aug 22;69(6):3285–99. doi: 10.1111/tbed.14680 (PMC10086952; doi:10.1111/tbed.14680)
Supplement: Supplementary file 3 — Supporting Material [file TBED-69-3285-s002.docx]

S1 Table. Number of aMPV molecular studies by order, genus and species, and total wild birds tested.

| **Order** | **Family** | **Genus** | **Species** | **No of studie**s | **Total tested** |
| --- | --- | --- | --- | --- | --- |
| Anseriformes | Anatidae | *Aix* | Wood duck (Aix sponsa) | 1 | 121 |
|  |  | *Anas* | Mallard (*Anas plathyrhynchos*) | 4 | 444 |
|  |  |  | American black duck (*Anas rubripes*) | 2 | 44 |
|  |  |  | Blue-winged teal (*Anas discors*) | 1 | 24 |
|  |  |  | Green-winged teal (*Anas carolinensis*) | 1 | 12 |
|  |  | *Mareca* | American wigeon (*Mareca americana*) | 1 | 1 |
|  |  | *Anser* | Greater white-fronted goose (*Anser albifrons*) | 1 | 224 |
|  |  |  | Greylag goose (*Anser anser*) | 1 | 30 |
|  |  |  | Bean goose (*Anser fabalis*) | 1 | 15 |
|  |  | *Aythya.* | Ring-necked duck (*Aythya collaris*) | 1 | 6 |
|  |  | *Branta* | Canada goose (*Branta canadensis*) | 3 | 556 |
|  |  |  | Barnacle goose (*Branta leucopsis*) | 1 | 72 |
|  |  | *Dendrocygna* | Black-bellied whistling duck (*Dendrocygna autumnalis*) | 1 | 5 |
|  |  |  | Fulvous whistling duck (*Dendrocygna bicolor*) | 1 | 1 |
|  |  |  | White faced whistling duck (*Dendrocygna viduata*) | 1 | 37 |
|  |  | *Cairina* | Muscovy duck (*Cairina moschata*) | 1 | 1 |
|  |  | *Spatula* | Northern shoveler (*Spatula clypeata*) | 1 | 5 |
| Charadriiformes | Scolopacidae | *Actitis* | Common sandpiper (*Actitis hypoleucos*) | 1 | 1 |
|  |  | *Arenaria* | Ruddy turnstone (*Arenaria interpres*) | 1 | 30 |
|  |  | *Calidris* | Dunlin (*Calidris alpina*) | 2 | 85 |
|  |  |  | Red knot (*Calidris canutus*) | 1 | 6 |
|  |  |  | Little stint (*Calidris minuta*) | 1 | 1 |
|  |  |  | Curlew sandpiper (*Calidris ferruginea*) | 1 | 3 |
|  |  |  | Ruff (*Calidris pugnax*) | 1 | 11 |
|  |  | *Tringa* | Common redshank (*Tringa totanus*) | 2 | 17 |
|  |  |  | Wood sandpiper (*Tringa glareola*) | 1 | 1 |
|  | Charadriidae | *Charadrius* | Common ringed plover (*Charadrius hiaticula*) | 1 | 5 |
|  |  | *Pluvialis* | Grey plover (*Pluvialis squatarola*) | 1 | 7 |
|  | Laridae | *Chroicocephalus* | Black-headed gull (*Chroicocephalus ridibundus*) | 1 | 18 |
|  |  | *Larus* | American herring-gull (*Larus smithsonianus*) | 1 | 24 |
|  |  |  | Great black-backed gull (*Larus marinus*) | 1 | 26 |
|  |  |  | Ring-billed gull (*Larus delawarensis*) | 1 | 7 |
|  |  |  | Common gull (*Larus canus*) | 1 | 84 |
|  |  |  | Mediterranean gull (*Larus melanocephalus*) | 1 | 53 |
|  |  |  | Yellow-legged gull (*Larus michahellis*) | 1 | 108 |
|  |  |  | European herring-gull (*Larus argentatus*) | 1 | 30 |
|  |  | *Sterna* | Common tern (*Sterna hirundo*) | 3 | 300 |
|  |  | *Sternula* | Little tern (*Sternula albifrons*) | 1 | 30 |
|  |  | *Thalasseus* | Sandwich tern (*Thalasseus sandvicensis*) | 1 | 86 |
|  | Haematopodidae | *Haematopus* | Eurasian oystercatcher (*Haematopus ostralegus*) | 1 | 18 |
| Columbiformes | Columbidae | *Columba* | Pigeon (*Columba livia*) | 2 | 18 |
| Falconiformes | Falconidae | *Falco* | American kestrel (*Falco sparverius*) | 1 | 2 |
| Galliformes | Phasianidae | *Phasianus* | Common pheasant (*Phasianus colchicus*) | 1 | 131 |
| Gruiformes | Rallidae | *Fulica* | American coot (*Fulica americana*) | 2 | 204 |
| Passeriformes | Passeridae | *Passer* | House sparrow (*Passer domesticus*) | 1 | 12 |
| Phoenicopteriformes | Phoenicopteridae | *Phoenicopterus* | Greater flamingo (*Phoenicopterus roseus*) | 1 | 35 |
| Piciformes | Ramphastidae | *Ramphastos* | Green-billed toucan (*Ramphastos dicolorus*) | 1 | 1 |
| Psittaciformes | *Psittacidae* | *Amazona* | Amazon parrot (*Amazona aestiva*) | 1 | 1 |
|  |  | *Psittacara* | White-eyed parakeet (*Psittacara leucophthalmus*) | 1 | 14 |
| Strigiformes | Strigidae | *Megascops* | Tropical screech owl (*Megascops choliba*) | 1 | 1 |

S2 Table. Number of aMPV serological studies by order, genus and species, and total wild birds tested.

| **Order** | **Family** | **Genus** | **Species** | **No of studie**s | **Total tested** |
| --- | --- | --- | --- | --- | --- |
| Anseriformes | Anatidae | *Branta* | Canada goose (*Branta canadensis*) | 1 | 310 |
| Charadriiformes | Laridae | *Chroicocephalus* | Black-headed gull (*Chroicocephalus ridibundus*) | 1 | 17 |
|  |  | *Larus* | American herring-gull (*Larus smithsonianus*) | 1 | 1 |
|  |  |  | Ring-billed gull (*Larus delawarensis*) | 1 | 13 |
|  |  |  | Mediterranean gull (*Larus melanocephalus*) | 1 | 50 |
|  |  |  | Yellow-legged gull (*Larus michahellis*) | 1 | 106 |
|  |  |  | European herring-gull (*Larus argentatus*) | 1 | 114 |
|  |  |  | Laughing gull (*Larus atricilla*) | 1 | 3 |
|  |  | *Sterna* | Common tern (*Sterna hirundo*) | 1 | 39 |
|  |  | *Sternula* | Little tern (*Sternula albifrons*) | 1 | 9 |
|  |  | *Thalasseus* | Sandwich tern (*Thalasseus sandvicensis*) | 1 | 69 |
| Columbiformes | Columbidae | *Columba* | Pigeon (*Columba livia*) | 1 | 194 |
|  |  | *Streptopelia* | Eurasian collared dove (*Streptopelia decaocto*) | 1 | 1 |
| Coraciiformes | Alcedinidae | *Megaceryle* | Belted kingfisher (*Megaceryle alcyon*) | 1 | 1 |
| Galliformes | Phasianidae | *Phasianus* | Common pheasant (*Phasianus colchicus*) | 2 | 415 |
|  | Numididae | *Numida* | Helmeted guineafowl (*Numida meleagris*) | 1 | 17 |
| Gruiformes | Rallidae | *Fulica* | American coot (*Fulica americana*) | 1 | 114 |
| Passeriformes | Corvidae | *Corvus* | American crow (*Corvus brachyrhynchos*) | 1 | 51 |
|  |  |  | Fish crow (*Corvus ossifragus*) | 1 | 3 |
|  | Icteridae | *Quiscalus* | Common grackle (*Quiscalus quiscula*) | 1 | 14 |
|  | Turdidae | *Turdus* | American robin (*Turdus migratorius*) | 1 | 1 |
| Pelecaniformes | Ardeidae | *Bubulcus* | Cattle egret (*Bubulcus ibis*) | 2 | 40 |
|  |  | *Ardea* | Great blue heron (*Ardea herodias*) | 1 | 1 |
|  |  |  | Great egret (*Ardea alba*) | 1 | 7 |
|  |  | *Egretta* | Little egret (*Egretta garzetta*) | 1 | 21 |
| Phoenicopteriformes | Phoenicopteridae | *Phoenicopterus* | Greater flamingo (*Phoenicopterus roseus*) | 1 | 30 |
| Strigiformes | Strigidae | *Bubo* | Great horned owl (*Bubo virginianus*) | 1 | 3 |
